# Recent advancements in targeted protein knockdown technologies—emerging paradigms for targeted therapy

**DOI:** 10.37349/etat.2023.00194

**Published:** 2023-12-26

**Authors:** Mansi Joshi, Pranay Dey, Abhijit De

**Affiliations:** Johannes-Gutenberg University of Mainz, Germany; ^1^Molecular Functional Imaging Lab, Advanced Centre for Treatment Research and Education in Cancer (ACTREC), Tata Memorial Centre, Kharghar, Navi Mumbai 410210, India; ^2^Life Science, Homi Bhabha National Institute, Mumbai 400094, India

**Keywords:** Protein knockdown, post-translational modification, proteolysis targeting chimeras, intrabodies, tripartite motif-21, cancer

## Abstract

A generalized therapeutic strategy for various disease conditions, including cancer, is to deplete or inactivate harmful protein targets. Various forms of protein or gene silencing molecules, e.g., small molecule inhibitors, RNA interference (RNAi), and microRNAs (miRNAs) have been used against druggable targets. Over the past few years, targeted protein degradation (TPD) approaches have been developed for direct degradation of candidate proteins. Among the TPD approaches, proteolysis targeting chimeras (PROTACs) have emerged as one of the most promising approaches for the selective elimination of proteins via the ubiquitin-proteasome system. Other than PROTACs, TPD methods with potential therapeutic use include intrabody-mediated protein knockdown and tripartite motif-21 (TRIM-21) mediated TRIM-Away. In this review, protein knockdown approaches, their modes of action, and their advantages over conventional gene knockdown approaches are summarized. In cancers, disease-associated protein functions are often executed by specific post-translational modifications (PTMs). The role of TRIM-Away is highlighted in the direct knockdown of PTM forms of target proteins. Moreover, the application challenges and the prospective clinical use of TPD approaches in various diseases are also discussed.

## Introduction

Proteins execute nearly all the functions in a living cell. Consequently, protein dysfunction leads to the development of various disease conditions, including neurological and immunological disorders, and cancer. Thus, approaches that can directly alter a target protein expression inside a cell provide unique opportunities to determine the function of that protein and develop new therapeutic intervention procedures. A common approach for modulating cellular protein function is targeting genetic alterations. Traditional methods for genome editing using either clustered regularly interspersed short palindromic repeats (CRISPR) and CRISPR associated proteins (Cas) system (CRISPR-Cas)-mediated knockout or RNA interference (RNAi)-mediated knockdown approaches are commonly used. CRISPR (CRISPR DNA sequences) gene-editing technology comprises an endonuclease with specific DNA-targeting activity and a guide RNA, that guides complete disruption of the targeted gene segment. The CRISPR-Cas9 system has several advantages over other gene-editing technologies, including higher specificity, feasibility, and low cost [1]. Whereas, using the RNAi method, gene silencing can be achieved by degrading the target gene transcript. Advantages of using RNAi include higher efficiency and ease of use, and it can be modulated for both short- and long-term silencing effects on the target molecule [2]. Despite remarkable progress in RNAi and CRISPR methodologies, several limitations were realized in their applications. First, both approaches provide an indirect method for protein depletion and therefore suffer from off-target effects, leading to undesired disruption of gene function [3]. Furthermore, long-term protein manipulation by these methods may result in the activation of other compensatory pathways or spontaneous mutations due to genomic alterations, which may hide the actual phenotypes [4, 5].

In addition to gene-level regulation, post-translational modifications (PTMs) also dictate protein functions in various disease conditions. In the PTM process, various functional groups are attached to specific amino acid residues, such as phosphorylation, methylation, and acetylation, etc. The addition of these moieties triggers cellular function by modulating protein folding, stability, and conformation [6]. As these intrusive properties of a protein define its functional state, the deregulation of certain target protein PTMs or their crosstalk with others contributes to various pathological conditions, including cancer onset and malignancy [7]. Also, many times, specific PTM of a protein might be responsible for the diseased condition, while other PTM forms of the same target contribute to normal cellular functions [8]. Therefore, direct knockdown of disease-specific PTMs instead of the gene or transcript may provide a unique opportunity to achieve precision control on protein function and develop novel therapeutics with reduced toxicity. Various new direct protein and/or PTM knockdown methods have been researched over the past years. These methods showed promise in achieving the goal without interfering with the gene or transcript expression (Table 1). Additionally, recent advancements in targeted protein degradation (TPD) technology have shown that controlling a specific PTM target is possible, leaving the total or other PTM forms of that target molecule intact.

**Table 1:** Advantages and limitations of different protein silencing techniques

**Technology**	**Target**	**Advantages**	**Disadvantages**
RNAi	RNA 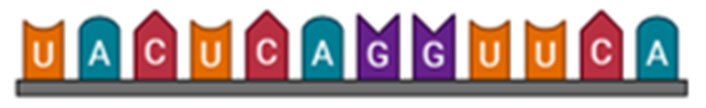	Can be used for either short-term or long-term silencing effect Supports both synthetic or genetic delivery methods to target cell Cost-effective and easy to use Can be used for inducible genetic knockdown	High off-target effect Mostly provide partial knockdown of target Activation of compensatory pathway Not effective for proteins with long half-life
CRISPR-Cas9	DNA 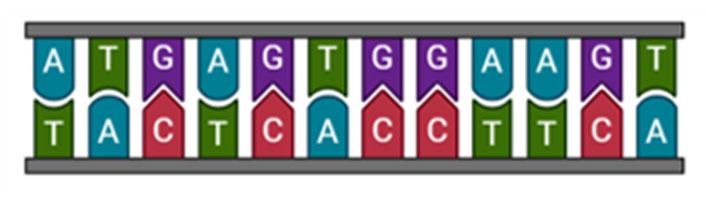	Gene editing with a complete knockout of a gene or a gene segment Precision Irreversible process High feasibility	Time-consuming Essential gene knockouts are lethal Difficult to aim larger proteins Off-target effect generating indels
Targeted protein knockdown	Protein 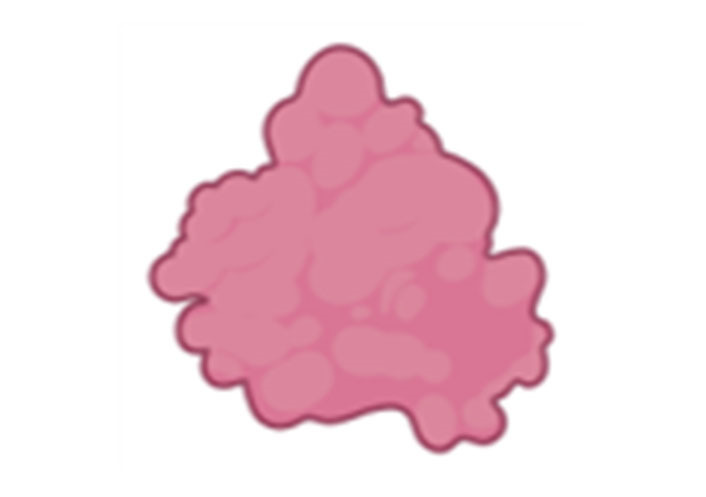	Directly eliminates target protein Can target essential or undruggable proteins Rapid, hence low chances of compensatory activation Less chance of immune response	Tedious design and synthesis challenge Expensive

Cells maintain protein homeostasis through a large number of interconnected pathways that regulate protein synthesis, conformation, cellular localization, and degradation. The disposal of misfolded or damaged proteins is performed by either a proteasomal-mediated pathway or a lysosomal-mediated pathway. The ubiquitin-proteasomal system plays a vital role in protein homeostasis by maintaining the turnover and thereby regulating crucial cellular processes, such as cell cycle, signal transduction, and transcriptional regulation [9]. Whereas, the lysosomal pathway includes protein degradation by endocytosis, phagocytosis, or by autophagy. Several TPD technologies have been developed based on lysosomal-mediated protein degradation. For example, Lysosome-Targeting Chimaera (LYTAC), antibody-based proteolysis targeting chimeras (PROTACs, AbTAC), autophagy-targeting chimera (AUTAC), and AUTOphagy-TArgeting Chimera (AUTOTAC) were synthesized for protein degradation purposes using either lysosomal-mediated endocytosis or autophagy [10]. Current research approaches that have helped in achieving the expansion of TPD approaches primarily include PROTAC technology, Intrabodies, and tripartite motif (TRIM)-Away. Most of the protein knockdown mechanisms such as using PROTACs or specific and nongenetic IAP-dependent protein erasers (SNIPERs), and TRIM-Away facilitate protein degradation via the ubiquitin-proteasome pathway. Whereas the intrabodies block the protein functions by neutralizing them intracellularly or they can also divert the proteins for ubiquitin proteasome-mediated degradation [11]. In this review, recent advancements in these protein knockdown strategies, and their future clinical perspectives are discussed.

## PROTACs

**Figure 1:**
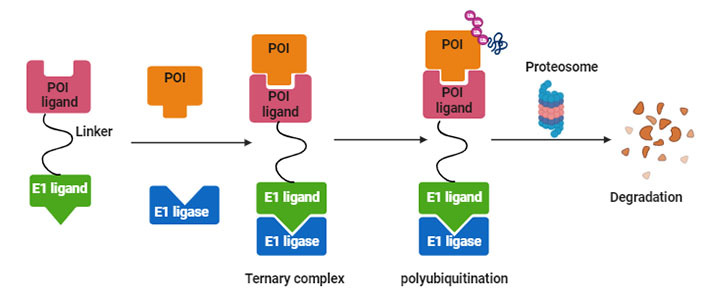
Systematic representation of PROTAC-mediated protein knockdown. The first step is the synthesis of PROTAC molecule, which includes a ligand for the protein of interest (POI) and ubiquitin E3 enzyme. In the next step, the PROTAC molecule binds with POI and E3 ligase to form a ternary complex, which eventually leads to polyubiquitination and subsequent degradation of POI via the ubiquitination pathway. Created with BioRender.com

**Figure 2:**
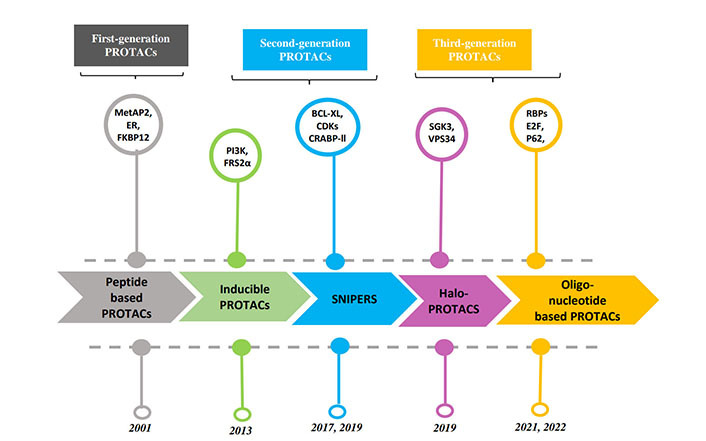
Timeline of advancement in PROTAC technology. MetAP2 [17]; FKBP12 [18, 19]; PI3K, FRS2α [20]; BCL-XL [25]. SGK3: glycose synthase kinase-3; VPS34: vascular protein sorting-34 [26, 27]; AR: androgen receptor; E2F: E2 transcription factor; RBPs: ribosome binding proteins [28]; P62: protein 62 [29]

PROTACs are a class of molecules that allow direct protein knockdown via the proteasomal degradation pathway. While traditional targeted drugs require strong binding affinity for the target protein, agents such as PROTACs can label a target protein through weak binding, thus offering a potential solution for 80% of the “undruggable” targets [12, 13]. PROTACs are hetero-bifunctional molecules that contain two ligands connected by a linker: one for recruitment and binding to the target protein, and the other for recruitment and binding to E3 ubiquitin ligase (Figure 1). Majorly, ligands used for E3 ligase are von Hippel-Lindau (VHL) and cereblon (CRBN), which are the components of the ubiquitin proteasome system (UPS). These ligands have several advantages over other E3 ligands, such as (1) strong affinity towards the ligands; (2) detailed information about their binding properties; and (3) admissible biophysical properties. Apart from PROTAC ligands [14], the composition and length of the linker play a critical role in determining the potency and selectivity of PROTACs. Depending on the target protein, different linker lengths and compositions may be required to achieve optimal activity [15]. Additionally, the location of linker attachment sites on the ligands can affect the degradation efficiency. Generally, the linker attachment sites are chosen to be in the regions of ligands that are exposed to solvents. The linker can be composed of various chemical moieties, such as amide bonds, carbon atoms, heteroatoms, etc., depending on the specific requirements of PROTAC design [16]. Since the discovery of the first PROTAC molecules, three successive generations of PROTAC molecules have been developed for various targets (Figure 2), which are summarized below.

### Types of PROTAC

#### Peptide-based PROTAC molecules

The first successful *in vitro* use of PROTAC technology was reported in 2001 to degrade methionine aminopeptidase 2 (MetAP2), a target of angiogenesis inhibitors in *Xenopus levis* eggs [17]. A peptide-based PROTAC-1 molecule was developed containing a domain recognizing MetAP2, and another domain recruiting MetAP2 to Skp-1Cullin-F-box complex (SCF, Skp-1 is a Cullin-F-box complex containing Hrt1) that in turn catalyzes the attachment of Ub to MetAP2. However, such peptide-based PROTACs showed membrane permeability issues, limiting their use as chemical probes. To overcome this problem, cell-permeable PROTACs were synthesized by adding a poly-D-Arg sequence to an E3 ligase complexed with VHL binding peptide sequence from hypoxia-inducible factor-1α (HIF-1α), which showed degradation of a range of target proteins, such as F506 binding protein 12 (FKBP12) F36V mutant protein and estrogen receptor [18, 19].

#### Inducible PROTACs

To achieve conditional protein knockdown, PROTAC having inducible activity upon tyrosine receptor kinase activation was reported as a second-generation PROTAC molecule. Nerve growth factor (NGF) binds to its receptor tropomyosin receptor kinase A (TrkA), which activates dimerization and auto-phosphorylation of TrkA. Phosphorylated tyrosine residues of TrkA bind to the phospho-tyrosine binding domain of fibroblast growth factor receptor substrate 2α (FRS2α) and activate downstream signaling. To block this signaling cascade, a phosphorylation-dependent PROTAC (TrkAPPFRS2α) was also synthesized consisting of a peptide sequence derived from TrkA. It has a central tyrosine residue for phosphorylation by TrkA, and another peptide derived from HIF-1α as a VHL ligand [20]. When NGF activates TrkA phosphorylated TrkAPPFRS2α PROTAC binds to FRS2α and inhibits the downstream signaling.

The first evidence of *in vivo* use of PROTACs was demonstrated by using a phosphoPROTAC (ErB2PPPI3K) molecule for induced knockdown of phosphoinositide 3-kinase (PI3K) protein when stimulated by growth factors. In addition to the poly-D-Arg and VHL binding ligands, phosphoPROTAC consists of a conditional protein binding ligand that recruits the target protein only when a growth factor is added to the cells. This phosphoPROTAC (ErB2PPPI3K) consists of a peptide sequence from erythroblastosis oncogene B3 [ErbB3, or epidermal growth factor receptor 3 (EGFR3)], which has a phosphorylation site that provides a binding site for PI3K, a downstream signaling component. Upon neuregulin stimulation, both ErbB3 and ErbB3 phosphorylation sites on PROTAC are phosphorylated, leading to recruitment, ubiquitinylation, and subsequent degradation of PI3K, and thereby reduced tumor growth by 40% in a murine model with no apparent side effects [20].

#### SNIPERs

Other second-generation PROTAC molecules have been developed for proteins such as VHL, CRBN, and inhibitors of apoptosis (IAP) ligands, which can be used as potential therapeutic agents in disease conditions. IAPs represent a class of negative regulators in the intrinsic apoptotic pathway that inhibit caspase action. IAP overexpression has been reported in various cancers and is associated with poor prognosis. Thus, IAPs are considered key targets for cancer therapy. IAP-based PROTACs are known as SNIPERs (specific and non-genetic IAP-dependent protein erasers). Because IAPs belong to the E3 ubiquitin ligase family, inhibitors of IAPs are generally used as ligands for E3 ligase [21]. Initially, the first-generation SNIPERs were made by using a ligand for POI and bestatin (an aminopeptidase inhibitor), a ligand for IAP, which makes cancer cells susceptible to apoptosis. The methyl-ester (MeBs) in bestatin binds with target protein cIAP, which subsequently gets recruited to the proteasome for degradation [22]. The successful use of first generation-SNIPERs mediated protein knockdown has been shown to knock various proteins like B-cell lymphoma-extra large (BCL-XL), B-cell lymphoma-Ableson (BCL-ABL), cyclin-dependent kinase (CDK), NOTCH1, retinoic acid receptor alpha (RARA), and estrogen receptor, which are further explained in the disease application section below. Even though these SNIPERs showed proficient knockdown of various targets, some limitations of using SNIPERs were also realized. First, as an aminopeptidase inhibitor, bestatin shows toxicity due to off-target binding. Second, SNIPERs show low efficacy because of which they are required at a higher concentration which makes them unsuitable for *in vivo* application. To overcome these challenges, second-generation SNIPERs with higher potency were generated, by replacing bestatin with MV1 (an IAP antagonist) as a ligand for the POI. Such SNIPERs showed higher potency than bestatin-associated SNIPERs for the successful degradation of various proteins, such as estrogen receptor α and CRABP-II [23, 24].

#### HaloPROTACs

Despite their success as TPD, both peptide and SNIPER-type PROTAC molecules showed low efficacy, requiring high concentrations of material to achieve maximal degradation. Addressing this problem, a new class of VHL-based PROTAC molecules (HaloPROTAC) was developed by creating HaloTag fusion proteins. This bi-functional, small molecule PROTAC ligand is synthesized with a binding propensity to the HaloTag7 (modified bacterial dehalogenase) that is fused to a POI of interest. The authors showed that by connecting a chlorinated alkane to the VHL ligand, the PROTAC can attach with the HaloTag7 fused to a green fluorescent protein (GFP). The other end of the PROTAC binds to the E3 ligase VHL and exerts ubiquitination of the GFP [25]. Owing to the HaloTag-mediated binding, this HaloPROTAC class of molecules showed improvements in specific targeting and degradation ability of the POI. The HaloPROTAC platform is therefore a potent tool for studying and validating protein functions. Later the use of the HaloPROTAC system was extended to target various endogenous proteins by combining it with CRISPR-Cas9 technology [26]. Using the HaloPROTAC-E (with modified VHL moieties) molecules, authors showed nearly complete degradation of tagged SGK3 and VPS34 proteins. Recently, a ligand-inducible, affinity-directed protein missile (L-AdPROM) system using HaloPROTAC (Halo-tag/VHL-recruiting PROTAC) was also developed as a method for tunable knockdown of POI [27].

#### Oligonucleotide-based PROTACs

Oligonucleotide-based PROTACs or O’PROTACs were designed to target RNA-binding proteins and transcription factors (TFs). These proteins play an essential role in DNA replication, repair, and transcription, and hence are associated with various disorders, such as cardiac and neuro-diseases, obesity, and malignancy. However, targeting these proteins remains challenging because they do not possess ligand-binding sites. To overcome this problem, various O’PROTACs, RNA-PROTACs, and TFs Targeting Chimeras (TRAFTACs) have been synthesized. O’PROTACs and TRAFTACs utilize a consensus double-stranded DNA (dsDNA) sequence to target TFs linked with a ligand for E3 ligase recruitment. Likewise, RNA-PROTACs utilize RNA consensus sequences for RBP-binding proteins linked with E3-ligase recruiting ligand [28]. The first report of a VHL-based TF-PROTAC molecule-mediated degradation was shown by targeting endogenous proteins E2F2 and p62 proteins [29]. Later, the TRAFTACs were developed which consist of a targeted-TF binding chimeric dsDNA sequence and a HaloTag-fused dCas9 protein-ligand for E3 ligase [30]. Using this approach effective degradation of brachyury (T-box TF family) and nuclear factor kappa-Β (NF-kB) was reported, suggesting potential use of the O’PROTACs for targeted degradation of RBPs and TFs.

### Disease applications

#### Application of PROTACs in cancer

Every year, nearly 2 million new cancer cases are reported worldwide, and it is a fatal disease for one in every six patients [31]. In recent years, cancer treatment has evolved to include targeted therapy and immunotherapy, alongside traditional approaches such as surgery, radiotherapy, and chemotherapy [32, 33]. The first-generation PROTACs showed that E3 ligase-mediated protein degradation can be used to modulate a target protein content inside a cell and thereby can be used as a potential strategy for cancer therapy. Therefore, the next step is to develop PROTACs as traditional drug-like small molecules, with improved stability both *in vitro* and *in vivo*, better biodistribution, and higher potency. To implement PROTAC-mediated approaches for cancer therapy, combinatorial ubiquitination real-time proteolysis (CURE-PRO) technology has been shown to reduce synthesis time and increase the efficiency for oncoprotein targets in the near future [34].

##### Breast cancer

Breast cancer is the most frequently diagnosed malignant neoplasm type among women worldwide, characterized by the uncontrolled growth and spread of cancer cells in other critical organs in the body. Prevention, diagnosis, and treatment for breast cancer have been an active area of research. Recently, to target poly(ADP-ribose) polymerase 1 (PARP1), researchers used E3 ubiquitin ligase murine double minute 2 (MDM2) ligand nutlin-3 derivative and PARP1 ligand niraparib derivative as a PROTAC, resulting in PARP1 degradation and apoptosis of human breast cancer cells MDA-MB-231 [35]. In another study, an IAP ligand derivative and an estrogen receptor ligand 4-hydroxytamoxifen were used to design a PROTAC, linked by alkyl and percutaneous endoscopic gastrostomy (PEG). This PROTAC showed induced estrogen receptor degradation, reactive oxygen species (ROS) production, and necrotic cell death in estrogen-dependent breast cancer cells. Focal adhesion kinase (FAK) is a critical scaffold for kinases and signaling proteins that play a crucial role in tumor invasion and metastasis. The traditional modulation of FAK activity with kinase inhibitors has limited success in clinical trials. The development of PROTACs as a novel strategy for FAK degradation has opened new possibilities. Crew’s group synthesized PROTAC 3, a FAK degrader, which demonstrated very good efficacy as compared to the FAK inhibitor defactinib in human triple-negative breast cancer (TNBC) cells [36]. Tanjoni et al. [37] synthesized a series of PROTACs based on CRBN ligand and FAK inhibitor PND-1186, linked by CRBN and PEG moieties that inhibited tumor growth in 4T1 and ID8 mice models. Their PROTAC also exhibited significant FAK target degradation in human pancreatic cancer cell line PA-TU-8988 T as well as PA-TU-8988 T xenograft models in nude mice [37]. These findings suggest that PROTAC-mediated FAK degradation is a promising therapeutic strategy for cancer treatment.

##### Hematological cancers

Acute myeloid leukemia (AML) is the most common acute leukemia in adults and is characterized by abnormal proliferation and impaired differentiation of hematopoietic precursor cells, leading to the accumulation of immature leukocytes in the bone marrow, peripheral blood, and other tissues. The advent of PROTACs has provided a promising approach to leukemia treatment. TRIM-containing protein 24 (TRIM-24) is a multi-domain protein involved in the transcriptional regulation of nuclear receptors, including AR. A PROTAC molecule was reported based on the TRIM-24 bromodomain inhibitor IACS-9571 and the VHL ligand [38]. This PROTAC recruited VHL to induce selective degradation of TRIM-24 in human AML MOLM-13 cells and thereby shows PROTAC as a new therapeutic avenue for targeting an “undruggable” protein target.

Excessive activation of CDKs results in dysregulated cell proliferation that promotes tumor progression, and inhibition of some CDK family members has proved as a viable approach to cancer therapy. However, the design of selective small-molecule inhibitors is hindered by similar ligand-binding pockets. Addressing this problem, a phthalimide-based PROTAC was designed which forms a different ternary complex with the E3 ligase CRBN and therefore exerts specific yet proteome-wide selective targeting of CDK6 [39].

The survival of T-cell acute lymphoblastic leukemia (ALL, T-ALL) cancer cells is dependent on the BCL-2 protein family member BCL-XL. However, the use of BCL-XL-specific inhibitors has been limited by their tendency to cause platelet toxicity and thrombocytopenia. However, a recent study showed that PROTAC designed for targeting BCL-XL can be a safe and effective adjuvant therapy for T-ALL. *In vitro* study results demonstrated high sensitivity of T-ALL cells to PROTAC and further *in vivo* T-ALL patient-derived xenotransplantation (PDX) models showed that a combination treatment of PROTAC and chemotherapy can reduce leukemia burden and prolong survival [40].

Recently, an interleukin-1 (IL-1) receptor-associated kinase-3 (IRAK3) PROTAC molecule was developed by conjugating an IRAK3 ligand and a CRBN ligand. The results showed that IRAK3 PROTAC effectively degraded more than 98% of IRAK3 in human monocytic leukemia THP1 cells and primary macrophages [41]. In chronic myelogenous leukemia (CML), the breakpoint cluster region-Abelson (BCR-ABL) fusion protein is a well-characterized causal agent, which is a result of chromosomal translocation [42]. While the development of BCR-ABL-targeted tyrosine kinase inhibitors (TKIs) has revolutionized CML treatment, researchers have attempted to develop PROTACs capable of inducing the degradation of the BCR-ABL fusion protein. The designed PROTAC molecules are composed of a kinase inhibitor ligand that binds to the ATP-binding site of BCR-ABL and a ligand that binds to E3 ubiquitin ligase. The CRBN-based PROTAC molecule has been shown to be effective in degrading both BCR-ABL and c-ABL receptors expression in K562 CML cells [43]. Additionally, BCR-ABL targeted ABL-SNIPER was also developed showing its ability to induce the target degradation in BCR-ABL-positive K562 cells and reduce cell growth [44]. Overall, these findings highlight the potential of PROTAC molecules as a novel and effective approach for the treatment of all types of hematological malignancies such as AML, T-ALL, and CML.

##### Colon cancer

In recent years, significant progress has been made in the field of cancer treatment through the targeted degradation of pathogenic proteins such as ERK, CDK9, bromodomain 4 (BRD4), and TRK. The first instance of a PROTAC-based application aimed at CDK9 protein as a target for human colorectal cancer. In the HCT116 cell line, this compound demonstrated dose-dependent degradation of CDK9, while the levels of CDK2 and CDK5 remained unaffected, highlighting the selectivity of the compound [45]. Another study has designed and synthesized two PROTACS for TRK [46]. These compounds are composed of CRBN E3 ligase-binding agents connected to TRK inhibitors, making them capable of targeting TRK proteins. In KM12 colon cancer cells, the two compounds led to a decrease in tropomyosin 3 (TPM3)-TRKA fusion protein levels as well as a decrease in AGBL carboxypeptidase 4 (AGBL4)-TRKB and ETS variant TF 6 (ETV6)-TRKC fusion protein levels [47]. These findings demonstrate the effectiveness of this approach in reducing the levels of pathogenic TRK proteins and may have potential applications in the treatment of colon cancer.

Even though PROTAC technology has emerged as a promising effective tool for drug discovery and therapy, it has certain disadvantages. One of which includes prior chemical synthesis of PROTAC molecules that makes the technique considerably complex. Also, PROTAC molecules show off-target effects and toxicity in clinical trials [48]. Therefore, discovering alternative approaches for TPD with more feasibility and specificity for target POI is needed.

#### Applications of PROTACS in other diseases

IRAK3 has been identified as a promising target for immune diseases, as it can inhibit pro-inflammatory signaling in innate leukocytes [49]. IRAK4 has emerged as a dual target for autoimmune diseases and cancer because of its crucial role in the immune response mediated by toll-like and IL receptors (ILRs). Another PROTAC molecule was designed to degrade histone deacetylase 3 (HDAC3) by linking the HDAC inhibitor anthranilide derivative and the CRBN ligand pomalidomide [50, 51]. The results of their study showed that PROTAC had minimal effect on gene expression in RAW 264.7 macrophages activated by lipopolysaccharide/interferon and thereby selectively downregulated HDAC3 levels.

Apoptosis signal-regulating kinase 1 (ASK1) is a widely expressed protein kinase that is redox-sensitive and involved in regulating apoptosis and signaling pathways such as inflammation and fibrosis under oxidative stress. Previous *in vivo* studies have demonstrated that inhibition of ASK1 can effectively reduce liver and kidney injury and fibrosis and may be a possible therapeutic target for non-alcoholic steatohepatitis (NASH) and diabetic kidney disease (DKD) [52]. Although the development of the ASK1 inhibitor GS4997 has progressed to phase III clinical trials, two of its key clinical trials for the treatment of NASH have failed, casting reservations on the development of drugs for this disease target. However, PROTAC technology offers potential use at low-dose for effective degradation of the target protein, making it a promising approach for targeting ASK1 and developing new treatments for diseases, such as NASH and DKD.

Tubulin-associated protein (Tau) is a microtubule-associated unit that plays a crucial role in axonal transport and microtubule stabilization within neurons [53]. Disrupted regulation of Tau protein has been implicated in several neurodegenerative diseases, including Alzheimer’s disease. Six small-molecule Tau-targeting PROTACs based on CRBN and VHL binders were developed, which effectively degraded Tau in human Tau-p301L and Tau-a152T neurons with favorable pharmacokinetic parameters [54]. The same group also synthesized novel Tau-targeting PROTACs, which preferentially degraded Tau in fronto-temporal dementia (FTD) neurons of frontotemporal dementia compared to normal cells [55].

The 3-hydroxy-3-methylglutaryl coenzyme A reductase (HMGCR) is a key enzyme in the cholesterol synthesis pathway and is a target of statins used to prevent and treat cardiovascular diseases. Luo et al. [56] reported a series of PROTAC molecules capable of degrading HMGCR in Chinese hamster ovary SRD15 cells. These PROTACs also activate the sterol regulatory element-binding protein pathway (SREBP) and inhibit cholesterol synthesis.

Research on PROTACs has gained excellent momentum as a promising and novel therapeutic modality because of their catalytic mechanism of action and potential clinical advantages. As of now, many PROTAC molecules are entering the early phase clinical trials as summarized in Table 2. They may exert excellent therapeutic indications where degradation of the whole protein is necessary and covalent inhibitors are insufficient or not selective enough. Some PROTACs are active at picomolar doses, making early research trials less risky. They may also be more selective than conventional small-molecule inhibitors because the active domain of the molecule is only required to bind to the target and not to elicit any changes in target function, unlike conventional inhibitors. Moreover, PROTACs may allow for tissue-specific target degradation, contingent on the tissue-specific expression of E3 ligases that PROTAC can leverage. However, the expandability of certain E3 ligases raises questions regarding whether cancer cells can lose dependence on E3 ligases and consequently become resistant to PROTAC.

**Table 2:** Summary of clinical trials for PROTACs

**Manufacturer**	**Compound name**	**Protein target**	**Disease**	**Ub ligase**	**Clinical trial stage**	**Clinical trial no.**
Arvinas	ARV-110	AR	Prostate cancer	CRBN	Phase II	NCT03888612
Arvinas/Pfizer	ARV-471	Estrogen receptor	Breast cancer	CRBN	Phase II	NCT05463952
Accutar Biotech	AC682	Estrogen receptor	Breast cancer	CRBN	Phase I	NCT05080842
Arvinas	ARV-766	AR	Prostate cancer	Undisclosed	Phase I	NCT05067140
Bristol Myers Squibb	CC-94676	AR	Prostate cancer	CRBN	Phase I	NCT04428788
Dialectic Therapeutics	DT2216	BCL-XL	Liquid and solid tumours	VHL	Phase I	NCT04886622
Foghorn Therapeutics	FHD-609	BRD9	Synovial sarcoma	Undisclosed	Phase I	NCT04965753
Kymera/Sanofi	KT-474	IRAK4	Autoimmune diseases (e.g., AD, HS)	Undisclosed	Phase I	NCT04772885
Kymera	KT-333	STAT3	Liquid and solid tumours	Undisclosed	Phase I	NCT05225584
C4 Therapeutics	CFT8919	EGFR-L858R	Non-small-cell lung cancer	CRBN	Pre-clinical	NA
Nurix Therapeutics	NX-5948	BTK	B cell malignancies and autoimmune diseases	CRBN	Phase I	NCT05131022

STAT3: signal transducer and activator of transcription 3; AD: atopic dermatisis; HS: hidradenitis suppurativa; NA: not applicable

Despite the promising potential of PROTACs, their development has been slow and the success rate in clinical studies has been limited. Currently, 71 PROTACs are developing in the oncology pipeline, with most of these agents still in the preclinical and discovery phases. So far, a spectrum of PROTACs are in different stages of clinical development. Two PROTACs, i.e. ARV-110 (bavdegalutamide) and ARV-766 are in phase II trial for metastatic castration-resistant prostate cancer (mCRPC). However, clinical trial results for ARV-471, a PROTAC targeting the estrogen receptor, have been disappointing. The drug yielded a lackluster 3% response rate in 71 heavily pre-treated estrogen receptor-positive/human EGFR 2 (HER2)-negative breast cancer patients in the VERITAC phase II single-arm trial investigating ARV-471 in combination with Pfizer’s CDK4/6 inhibitor, Ibrance (palbociclib). The primary endpoint was the clinical benefit rate, which occurred at a median rate of 38%. In summary, there are several caveats remaining limiting the development of PROTACs. Major factors include poor membrane permeability, oral bioavailability as well as insufficient evidence from human clinical studies. Nonetheless, the potential benefits of PROTAC technology make it a promising area of research and development for oncology and other disease areas.

## Intrabodies

Intrabodies are antibodies designed to be produced inside cells and target intracellular targets. There are two main subtypes of intrabodies: cytosolic/nuclear intrabodies and endoplasmic reticulum (ER) retained intrabodies. Cytosolic or nuclear intrabodies function by blocking targets through antibody-mediated neutralization, while ER intrabodies knockdown proteins of secretory pathways without neutralizing them. Also, ER intrabodies do not require specific selection for folding as these are produced inside the ER and bind with the antigen passing through the ER. Whereas, proper folding is required in reducing the environment of cytoplasm for cytosolic or nuclear intrabodies to bind with target antigens. Intrabodies provide very high specificity for the target protein and hold the ability to knock down multiple isoforms, splice variants, or even PTMs of the same target. Intrabodies are synthesized using different technologies such as phage display techniques, ribosomal display techniques, and yeast and bacterial display techniques. Complementary DNA (cDNA) for the target protein was cloned from the pre-existing antibody clone or by cloning the selected antibody domains (such as Fabs/scFv) using *in vitro* display techniques to provide more specificity. Previously, Kashima et al. [57] reported a novel cell-based intracellular protein-protein interaction detection platform (SOLIS) that enabled the screening of cytoplasmic intrabodies. In contrast, human or murine antibodies from some organisms, such as camels and sharks, possess single-domain antibodies (sdAbs) [58]. As the name suggests, these antibodies consist of a variable heavy chain only, while the light chain is absent. Liu et al. [59] reported that antibodies from sharks and camels show high levels of refolding ability and thermal stability owing to their smaller size. Although intrabodies are successfully produced in cells, not all intrabodies are correctly folded to reduce the intracellular environment, which interferes with the formation of disulfide bonds. This problem can be addressed by designing synthetic scaffolds, such as affibodies (peptides derived from protein A), designed ankyrin repeat proteins (DARPins), or fibronectin folds. These scaffolds do not require di-sulfide bonding and omit the additional requirement of functionality tests in the cytosol. The applicability and intracellular use of intrabodies are affected by their sizes and biochemical properties of cytoplasmic intrabodies.

Currently, cytosolic antibodies are generated for Tau proteins to understand their role in neurodegenerative disorders, and for cytoplasmic protein kinases, such as spleen tyrosine kinase (SYK), and EGFR [60–62].

### Delivery strategies

The current methods for delivering the intrabody genes are by transfection of cells by viral transduction, using encapsulation in nanoparticles, vaccines, peptides, or direct delivery of proteins. Owing to high transduction efficiency and low immunogenicity, adeno-associated viruses (AAVs) are the most promising tools for gene delivery. The first oncolytic viral immunotherapy, talimogene laherparepvec, was approved by the Food and Drug Administration (FDA) for the treatment of melanoma [63]. Encapsulation of intrabody genes in nanoparticles improved their stability and feasibility for intracellular delivery. The LNPLH messenger RNA (mRNA) complex (a cationic liquid nanoparticle and mRNA for the intracellular antibody complex) exhibited efficient delivery and low toxicity when introduced into different human cancer cell lines [64]. Cell-penetrating poly(disulfide)s (CPDs) were considered useful tools for the direct delivery of proteins. However, due to the constant positive charge in arginine-rich CPDs, there are limitations to the systemic delivery of intrabodies. This problem was hastened by the development of a pH-responsive CPD that delivers proteins into targeted tumor cells via the addition of protons to imidazole groups within the acidic tumor microenvironment (TME). Recently, a report has shown that in addition to intrabody gene delivery in mouse models, these genes can also be delivered *in vitro* to blood cells, which can again be delivered to the body [65]. Tisagenlecleucel and axicabtagene ciloleucel are approved for use in B cell ALL and large B cell lymphoma [66]. Both drugs are based on *ex vivo* delivery of intrabody genes into T cells, followed by re-introduction in patients.

### Therapeutic applications against virus infection, neurodegenerative disorders, and cancer

As intrabodies possess several advantages over conventional antibodies used against various disease-relevant target proteins, multiple clinical trials are ongoing using intrabodies (Table 3). The role of the viral infectivity factor (VIF) protein in the pathogenesis of human immunodeficiency virus type-1 (HIV-1) is crucial as it inhibits the antiviral activity of Apobec3G, which leads to enhanced viral replication and infectivity. Thus, targeting the VIF protein is a potential therapeutic approach for the treatment of HIV-1. A study highlighted a specific single-chain antibody against the VIF protein of HIV-1. The antibody was expressed intracellularly and inhibited reverse transcription and viral replication. To achieve this, a minimal VH fragment scaffold with intrabody properties derived from an anti-VIF single-chain antibody was engineered to mimic camelid antibody domains. The researchers showed that the VH single domains preserved antigen-binding activity and specificity in the absence of the parent VL domain. However, to prevent the non-specific binding of VH by its interface with the light chain variable domain, amino acid mutations in frameworks 2 and 4 were introduced (Val37F, G44E, L45R, W47G, and W103R). The best-folded domains had high levels of intracellular expression, indicating their potential as therapeutic agents. The expression of VH single-domains in eukaryotic cells showed that they could correctly fold as soluble proteins in a reducing environment. These results suggest that VH single domains targeting the VIF protein of HIV-1 have the potential to be developed as therapeutic agents for the treatment of HIV-1 [67].

Huntington’s disease (HD) is caused by a mutation in the huntingtin (HTT) protein that leads to protein misfolding and aggregation. Intrabodies can reduce HTT aggregation by binding to the toxic fragment and inactivating it. A single-domain intracellular antibody against HTT called V(L)12.3, has been engineered to inhibit aggregation and rescue toxicity in a neuronal model of HD as well as in a yeast model. V(L)12.3 is more potent than earlier anti-HTT intrabodies and may be a potential gene therapy for HD [68]. VH domain-containing protein (VHCP) intrabody targeting HTT was developed using VHCP, which specifically binds to the N-terminal region of the HTT. The intrabody was shown to reduce HTT aggregation *in vitro* and *in vivo*. A phase I/IIa clinical trial evaluating the safety, tolerability, and pharmacokinetics of this intrabody (IONIS-HTTRx) was conducted in patients with early-stage HD, and the results showed that the treatment was well tolerated.

Most monoclonal antibodies (mAbs) or their derivatives are used in cancer immunotherapy to target tumor antigens present on the cell surface, such as EGFR, HER2, vascular endothelial growth factor receptor 2 (VEGFR-2), and programmed death-ligand 1 (PD-L1). In contrast to targeted mAbs against cell surface markers, intrabodies can be used as both intracellular tumor-associated antigens (TAAs) and neoantigens. TAAs are generally present and expressed in both normal and tumor cells. However, the expression level is higher in tumor cells than in normal cells, thus providing a suitable clinical target. TAAs expressed on the cell surface are generally passed through the secretory pathways and are targeted by ER intrabodies. To generate cell-surface receptors targeting intrabodies using hybridomas, genes for VHL and VLL of the mAb are polymerase chain reaction (PCR)-amplified and then assembled and cloned into the vector with an ER-targeting sequence to retain the intrabodies in the ER. Along with targeting cell surface TAAs, ER intrabodies targeting membrane proteins in intracellular compartments have also been developed, such as intrabodies against toll-like receptor 9 (TLR9), which diminishes the development of pancreatic tumor [69]. Whereas, neo-antigens are specifically expressed by cancer cells due to somatic mutations, such as insertion-deletion (INDEL), and promote tumor development [e.g., mutated Rat sarcoma (RAS) proteins]. Different methods, such as DARPins, have been used to generate and select intrabodies against neoantigens. These intrabodies provide advantages over conventional antibodies to target HER2 and mutant RAS in both *in vitro* and *in vivo* tumor suppression [70, 71]. Additionally, a humanized antibody with a VH domain was developed to target the activated forms of WT-HRAS (wild type) and mutant HRAS (G12V) [72]. ER intrabodies against various oncogenic targets have shown inhibition of various oncogenic TAAs, such as EGFR, IL-2R, and metalloproteinases (MMPs), tested both *in vitro* and in preclinical mouse models.

Protein-tyrosine kinase (ETK, also called Bmx), the 70-kDa member of the Tec family of nonreceptor protein tyrosine kinases, is expressed in a variety of hematopoietic, epithelial, and endothelial cells. A previous study used a human single-domain antibody phage display library to generate intrabodies against ETK, a protein implicated in the proliferation, differentiation, and motility of cancer cells. The intrabodies were found to specifically bind to recombinant ETK and block its kinase activity. When expressed in transformed cells, the intrabodies led to the significant blockade of ETK enzymatic activity and inhibition of clonogenic cell growth in soft agar [73].

Researchers have generated single-domain antibodies against CapG, a constituent of the actin cytoskeleton overexpressed in breast cancer, and used them as intrabodies to inhibit the interaction of CapG with actin. One CapG nanobody was found to bind with nanomolar affinity to the first CapG domain and block the CapG interaction with actin. Expression of nanobody in breast cancer cells reduced cell migration and invasion phenotypes that resulted in the lower frequency of lung metastatic lesions in mice. The nanobody was also successfully delivered into cancer cells using bacteria harbouring a type III protein secretion system (T3SS) [74].

**Table 3:** Summary of important intrabodies in the preclinical phases of development

**Compound name**	**Protein target**	**Disease**	**Trial stage**
β42-Specific scFv	Amyloid-β	Alzheimer’s disease	Pre-clinical
Anti-α-syn—PEST nanobodies	α-Syn	Parkinson’s disease	Pre-clinical
Anti-HTT	Huntington	HD	Pre-clinical
EGFR-specific scFvs	EGFR	Epidermoid carcinoma	Pre-clinical
Anti-folate receptor	Folate receptor	Ovarian cancer	Pre-clinical
LL3 (anti-β-catenin intrabodies)	β-catenin	Familial adenomatous polyposis	Pre-clinical

α-Syn: α-synuclein; PEST: proteosome-targeting proline, aspartate or glutamate, serine, and threonine motif

### Challenges associated with intrabody-based therapeutics

#### Delivery

Intrabodies must be delivered efficiently to target cells or tissues. Large size and complex structure can hinder their ability to cross the cell membranes, thereby limiting their effectiveness [75].

#### Stability

Intrabodies need to remain stable *in vivo* to avoid degradation and maintain their binding activity. However, proteases and other enzymes in the body can degrade intrabodies, thereby nullifying their potential [76].

#### Immunogenicity

Intrabodies derived from non-human sources can be recognized as foreign antigens by the immune system, thereby reducing their effectiveness or causing adverse reactions [77].

#### Off-target effects

Intrabodies can bind to unintended targets, potentially causing adverse effects or interfering with normal cellular functions.

#### Cost

Producing intrabodies on a large scale for clinical use can be expensive and time-consuming, limiting their accessibility as therapy.

Overall, while intrabodies hold promise as a therapeutic modality, addressing these challenges is crucial for their successful translation to the clinic.

## TRIM-Away

Over the last five years or so, various studies have reported a new strategy of direct protein and PTM knockdown by utilizing intracellular TRIM-21 expression. TRIM-21 is a cytoplasmic protein that belongs to the E3 ubiquitin ligase family with a strong binding affinity to the Fc domain of antibodies. It consists of an N-terminal domain (NTD), a B-box domain, and a C-terminal domain (CTD). The NTD is responsible for E3 ubiquitin ligase activity, the B-box domain helps in dimerization, and the CTD consists of the antibody binding sequence, PRYSPRY, and regulates TRIM-21 function [78]. During infection, TRIM-21 recognizes antibody-bound pathogens, recruits them to the ubiquitin-proteasome system, and leads to their degradation. Apart from its natural physiological role in cellular defense, TRIM-21 has recently been utilized as a robust technique for the rapid and acute degradation of endogenous proteins in mammalian cells. The TRIM-Away methodology for TPD was reported, in which microinjecting an immunoglobulin G (IgG) against a specific protein into the cells overexpressing TRIM-21 led to specific degradation of the targeted protein in the cells. Successful knockdown of GFP post microinjection of anti-GFP antibody into NIH 3T3 cells overexpressing TRIM-21 and free GFP was shown using TRIM-Away [79]. In addition, when GFP was tagged with endogenous proteins and localized to different organelles, such as the nucleus, plasma membrane, and endosomal membranes within primary oocytes of mice, GFP-tagged proteins were efficiently degraded by the TRIM-Away approach. However, the degradation of nuclear proteins requires higher antibody concentration in the nucleus due to the large size of used antibodies that hinders their nuclear localization. This problem was overcome by using an Fc fragment tagged with an anti-GFP nanobody, a smaller antibody form that improved its nuclear transport and degradation efficiency. TRIM-21-mediated knockdown of a stable protein REC8 meiotic recombination protein (Rec8) in mouse eggs demonstrated its effectiveness over standardized gene knockdown approaches, such as siRNA that cannot effectively degrade such long-lived proteins due to their slow turnover rate. In addition to that, depletion of nucleotide-binding domain, leucine-rich-containing family, pyrin domain-containing-3 (NLRP3) and inhibitor of nuclear factor-kB kinase (IKK) proteins in primary human cells (human macrophages) was also reported that led to the functional alterations in macrophages and reduced their production of pro-inflammatory cytokines, such as IL-1ꞵ. Lately, Nag et al. [80] elaborated an unidentified role of TRIM-21 in regulating human antigen R (HuR), an RNA-binding protein, required for regulating cell survival under stress conditions. The authors found that under heat-shock conditions AKT1 phosphorylates HuR at the K182 position, priming it for TRIM-21 mediated ubiquitination. This enables a cellular adaptive mechanism that aids in cell survival.

**Figure 3:**
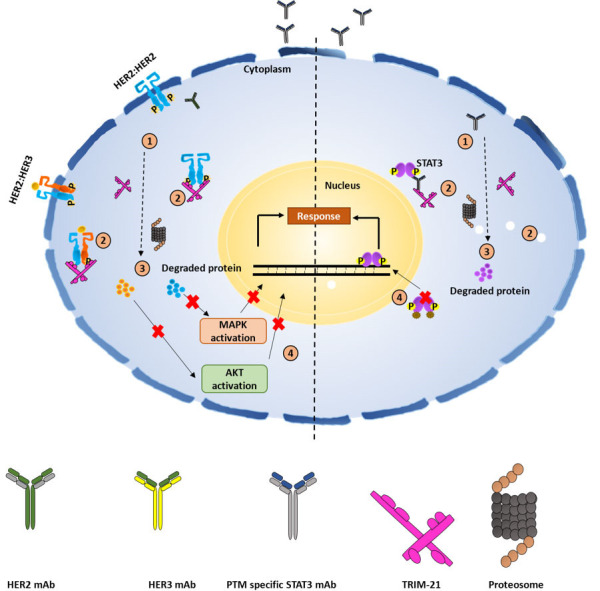
Schematic representation for TRIM mediated protein knockdown of membranous protein HER2 and HER3, and specific PTM forms of cytosolic protein STAT3. 1. Transfection of mAbs specific for targeted protein in breast cancer-TRIM-21 overexpressing cells; 2. formation of TRIM-21-antibody-protein complex; 3. proteasomal degradation of targeted proteins; 4. knockdown of HER2, HER3 and STAT3 protein leads to inactivation of the downstream signaling respectively. P: phosphorylation

Although the TRIM-AWAY technology seems intriguing and easy to use, the technique described was laborious and expensive. Further, the therapeutic implications of degrading disease-relevant target proteins were not elaborated. To address these conundrums, we utilized a distinctive methodical framework in our study like electroporation and mAb transfection to deliver the required mAbs in breast cancer cell population. This led us to discover promising onco-therapeutic advancements for breast cancer by targeting two specific oncoproteins of interest. Notably, we presented evidence for the first time that activated forms of HER2 and HER3 receptor proteins as well as cytoplasmic STAT3 phospho-PTMs forms in breast cancer cells can be directly targeted using our approach, which was referred to as the “TRIM-ing” process (Figure 3). Taking advantage of this method, we further demonstrated selective degradation of the phospho-PTM form of the STAT3 target by using phospho-PTM-specific mAbs. STAT3 protein shows its downstream signaling post-activation by phosphorylation at tyrosine residue (Y705) and /or serine residue (S727). We have shown that the TRIM-ing of pY705-STAT3 decreased the expression of only the pY705 PTM form, without affecting pS727-STAT3 levels and vice-versa [81].

In immune cells, binding of TRIM-21 with antigen-coated antibodies induces a signaling cascade and innate immune response. However, overexpression of TRIM-21 in targeted cells does not show any significant changes in the expression levels of its conventional signaling partners, such as interferon regulatory factor-3 (IRF-3), sequestosome (SQSTM), IRF-5, DEAD-box protein 41 (DDX41), and Skp2, suggesting cells artificial overexpression of TRIM-21 does not affect cellular phenotype [81, 82]. When combined with chemotherapeutic agents for HER2-positive breast cancer (neratinib or trastuzumab), HER2-TRIM-ing enhanced drug efficacy and the levels of downstream molecules, p-ERK and p-AKT further decreased, validating the therapeutic potential of TRIM-ing technology. One of the limitations of using HER2-targeted inhibitors is the activation of compensatory pathways, such as EGFR or HER3 activation. However, post-TRIM-ing, no compensatory RTK activation was observed for HER2 or HER3 targets, proving the novel therapeutic efficacy of TRIM technology. In addition to membranous proteins, TRIM-ing of specific PTM forms of STAT3 also resulted in similar cytotoxic effects in breast cancer cells alone, which was further synergized with STAT3 inhibitors like stattic or niclosamide.

### Delivery methods

The efficiency of TRIM-Away depends on the antibody delivery methods for both *in vitro* and *in vivo* applications. To achieve TRIM-Away in *Xenopus* eggs, *trim21* encoding mRNA with or without antibody was microinjected in embryos. While microinjection is suitable for antibody delivery in individual cells, antibody electroporation can also be used to efficiently deliver antibodies in wide varieties of cell populations. Also, electroporation-mediated antibody delivery at optimized conditions does not exhibit significant stress on the cells [79]. However, the large size of some conventional antibodies limits their accessibility to the cells and decreases the TRIM-ing efficacy of target proteins. To address this problem, TRIMbody was reported by fusing smaller formats of antibodies, called nanobodies, with a TRIM of TRIM-21 [82]. They synthesized TRIMbody to target enhanced GFP (EGFP), using a bispecific anti-EGFP nanobody which showed efficient degradation of endogenous EGFP *in vitro*. Also, Du et al. [83] reported a mix-and-go approach for delivering antibodies inside the cells by using CPDs that showed successful TRIM-21-mediated degradation of various targets [83]. Recently, another technology called cell-free TRIM-Away was developed, demonstrating the potential application of TRIM21-mediated knockdown to understand protein functions in cell-free systems [84]. The cell-free TRIM-Away was performed for TFIIS (a transcription elongation factor) and nuclear protein nuclearization protein 4 (NPL4) degradation, in antibody and TRIM-21 containing supernatant extracts isolated from *Xenopus* eggs. In contrast to cell-based TRIM-Away, cell-free TRIM-Away required around 50-fold less antibody and complete degradation of targeted proteins was achieved within a few minutes. Indeed, these methods provide a successful approach for TPD both in cells and embryos, further refinements of these methodologies may be required for targeting proteins or specific PTM marks *in vivo*.

### Disease application

As proteins play a central role in biological functions, genetic mutations often lead to aberrant expression and activation of various proteins, involved in disease development. Cancer and neurodegenerative diseases are prime examples of diseases caused by mutated proteins. TRIM-Away provides a specific knockdown of target proteins that can be used as a powerful tool as a therapeutic strategy to target disease-causing proteins. In HD, a neurodegenerative disorder, the mutated HTT protein disrupts normal functions of brain cells and leads to neurodegeneration over time. Successful degradation of this mutated form of the HTT protein was done using TRIM-Away while preserving the wild-type HTT protein [79]. Further, TRIM-ing of oncogenic proteins HER2 or HER3 alone showed inhibition of their downstream signaling cascade, i.e. ERK activation, and AKT activation, respectively, leading to cell death in HER2-overexpressing overexpression has been reported in breast cancer cells. For metastatic colon cancer, targeting TRIM-21 with vilazodone activates UPS-mediated degradation of yes activating protein (YAP) and exhibits anti-metastasis effects [85]. A recent report shows that TRIM-21-mediated polyubiquitination and degradation of severe acute respiratory syndrome coronavirus 2 (SARS-CoV-2) proteins and inhibits virus particles assembly by using host proteasome system and may be applied to develop novel therapeutic strategies or SARS-CoV variants [86].

## Conclusions

Targeted protein knockdown is an attractive approach that is being actively pursued as a therapeutic strategy. Here we have summarized the advantages and limitations of major targeted protein knockdown approaches, including PROTACs-mediated knockdown, intrabodies-mediated knockdown, and TRIM-Away (Table 4). There are several important strategies developed already for selective and rapid degradation of target proteins of interest as opposed to the conventional gene knockdown or knockout approaches. Compared to small-molecule inhibitors, TPD provides advantages such as (1) selectivity and specificity; (2) degradation of non-pharmacological targets; (3) overcoming drug resistance; and (4) targeted knockdown of specific PTMs. Considering these advantages and recent advancements in developing methodologies discussed here, TPD could emerge as a potential therapeutic option for various disease conditions, including cancers.

**Table 4:** Advantages and limitations of different protein knockdown approaches

**Method**	**Advantages and limitations**
**PROTAC** 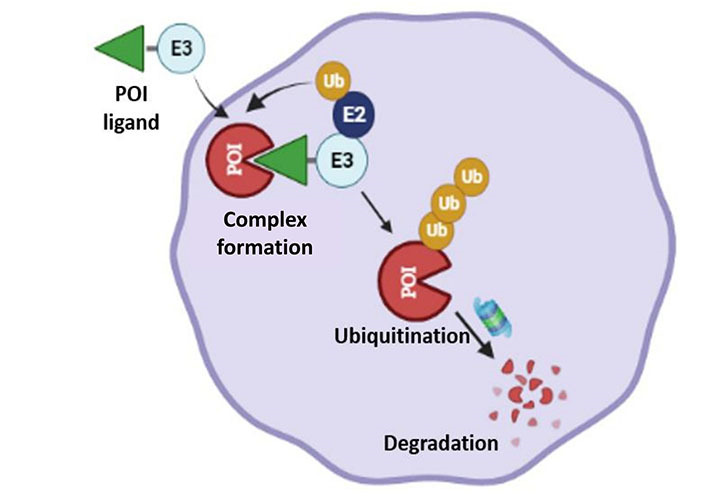	**Advantages:** Can be used for undruggable molecules Targeted degradation Low toxicity Avoids development of drug resistance **Limitations:** Requires prior chemical synthesis Off-target activity Only for intracellular protein
**Intrabodies** 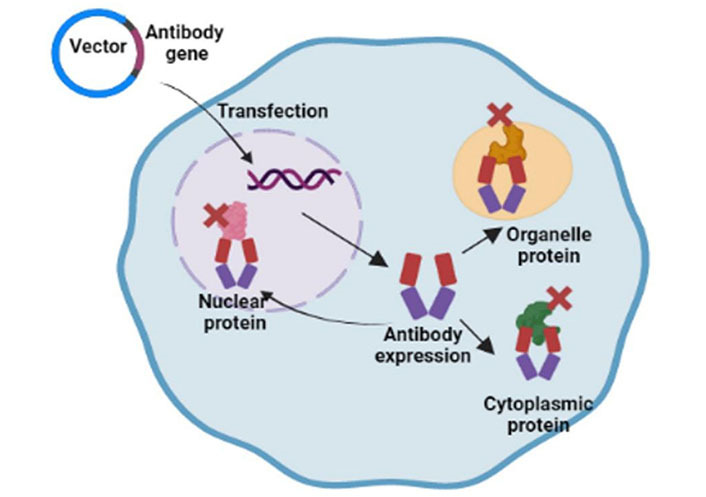	**Advantages:** More specific Can target splice variants or mutant forms For both membranous and intracellular proteins **Limitations:** Time-consuming Off-target effects Immunogenicity Needs modifications for netter delivery and stability
**TRIM-Away** 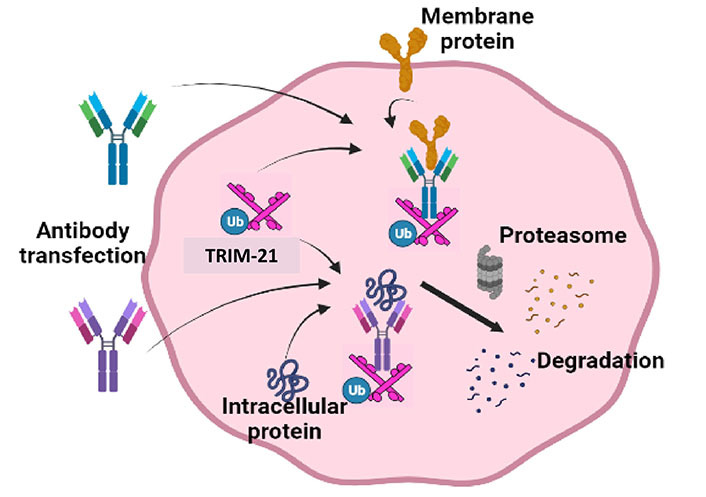	**Advantages:** High specificity Rapid and easy to use Does not require prior chemical synthesis Can target specific PTMs **Limitations** Require highly-specific antibodies Lack of in vivo validation Requires exogenous delivery of antibody

TPD can be achieved either by utilizing a proteasomal-mediated pathway or a lysosomal-mediated pathway. PROTACs, such as SNIPERs and IAP-based PROTACs have shown promise to degrade various undruggable targets by using the UPS. Additionally, another TPD methodology called TRIM-Away has emerged, which also relies on UPS providing an opportunity for rapid degradation of POI. Unlike the genetic knockdown approaches, TRIM-Away provides the ability to shut down specific phospho-PTM or other activated forms of a target protein. Both PROTACs and TRIM-Away have shown promising results in pre-clinical settings. Whereas, lysosomal-mediated protein degradation methods are still in the early developmental stage, and the mechanism of action for lysosomal-meditated protein knockdown technology is not clear yet. Also, as lysosomes are vital organelles that regulate enormous functions in the cell, lysosomal hijacking leads to an adverse effect *in vivo*, which may require careful consideration. As only a few targets have been identified including asialoglycoprotein (ASGPR) and cation-independent mannose-phosphate receptor (CI-MPR), additional lysosome-targeting receptors are needed to be identified to expand the lysosomal-mediated pathway for a larger protein-spectrum, as available for UPS-based technologies, such as PROTACs.

In the last few years, various PROTAC molecules have successfully reached phase I and II trials for cancer treatment. The TRIM-Away methodology has also emerged as a powerful technique to understand protein functions and may emerge as a novel therapeutic approach in the future. The other approach for direct reduction of the target protein is by using antibodies against POI to neutralize them and inhibit their function. Intrabodies are synthesized to inhibit TAAs and neoantigens present at intracellular locations, which are otherwise difficult to target using conventional full-length antibodies. These methodologies have shown their promise in shutting down the specific activated form of a target protein, without affecting the total/unmodified protein and thus provide the limited chance of compensatory pathways activation. Overall, it is expected that further refinement and innovative research efforts will continue along the line of direct protein knockdown approaches and eventually, that will bring in novel therapeutics for the clinic.

## Abbreviations

AML: acute myeloid leukemia
AR: androgen receptor
ASK1: apoptosis signal-regulating kinase 1
BCL-XL: B-cell lymphoma-extra large
BCR-ABL: breakpoint cluster region-Abelson
Cas: clustered regularly interspersed short palindromic repeats associated proteins
CDK: cyclin-dependent kinase
CML: chronic myelogenous leukemia
CPDs: cell-penetrating poly(disulfide)s
CRBN: cereblon
CRISPR: clustered regularly interspersed short palindromic repeats
EGFP: enhanced green fluorescent protein
EGFR3: epidermal growth factor receptor 3
ER: endoplasmic reticulum
ErbB3: erythroblastosis oncogene B3
ETK: protein-tyrosine kinase
FAK: focal adhesion kinase
FKBP12: F506 binding protein 12
GFP: green fluorescent protein
HD: Huntington’s disease
HDAC3: histone deacetylase 3
HER2: human epidermal growth factor receptor 2
HIV-1: human immunodeficiency virus type-1
HTT: huntingtin
IAP: inhibitors of apoptosis
IL-1: interleukin-1
IRAK3: interleukin-1 receptor-associated kinase-3
mAbs: monoclonal antibodies
MetAP2: methionine aminopeptidase 2
mRNA: messenger RNA
NASH: non-alcoholic steatohepatitis
PARP1: poly(ADP-ribose) polymerase 1
PI3K: phosphoinositide 3-kinase
POI: protein of interest
PROTACs: proteolysis targeting chimeras
PTMs: post-translational modifications
RNAi: RNA interference
SGK3: glycose synthase kinase-3
SNIPERs: specific and nongenetic IAP-dependent protein erasers
STAT3: signal transducer and activator of transcription 3
TAAs: tumor-associated antigens
T-ALL: T-cell acute lymphoblastic leukemia
Tau: tubulin-associated protein
TFs: transcription factors
TPD: targeted protein degradation
TRAFTACs: Transcription Factors Targeting Chimeras
TRIM-21: tripartite motif-21
TrkA: tropomyosin receptor kinase A
UPS: ubiquitin proteasome system
VHL: von Hippel-Lindau
VIF: viral infectivity factor
VPS34: vascular protein sorting-34

## Declarations

### Copyright

© The Author(s) 2023.
